# Mu Opioid Receptor Binding Correlates with Nicotine Dependence and Reward in Smokers

**DOI:** 10.1371/journal.pone.0113694

**Published:** 2014-12-10

**Authors:** Hiroto Kuwabara, Stephen J. Heishman, James R. Brasic, Carlo Contoreggi, Nicola Cascella, Kristen M. Mackowick, Richard Taylor, Olivier Rousset, William Willis, Marilyn A. Huestis, Marta Concheiro, Gary Wand, Dean F. Wong, Nora D. Volkow

**Affiliations:** 1 The Russell H. Morgan Department of Radiology and Radiological Science, Johns Hopkins University, Baltimore, United States of America; 2 Department of Psychiatry, Johns Hopkins University, Baltimore, United States of America; 3 Department of Medicine, Johns Hopkins University, Baltimore, United States of America; 4 Department of Neuroscience, Johns Hopkins University, Baltimore, United States of America; 5 Nicotine Psychopharmacology, National Institute on Drug Abuse, Intramural Research Program, Baltimore, United States of America; 6 Chemistry and Drug Metabolism Sections, National Institute on Drug Abuse, Intramural Research Program, Baltimore, United States of America; 7 National Institute on Drug Abuse, Rockville, United States of America; Tokyo Metropolitan Institute of Medical Science, Japan

## Abstract

The rewarding effects of nicotine are associated with activation of nicotine receptors. However, there is increasing evidence that the endogenous opioid system is involved in nicotine's rewarding effects. We employed PET imaging with [^11^C]carfentanil to test the hypotheses that acute cigarette smoking increases release of endogenous opioids in the human brain and that smokers have an upregulation of mu opioid receptors (MORs) when compared to nonsmokers. We found no significant changes in binding potential (BP_ND_) of [^11^C]carfentanil between the placebo and the active cigarette sessions, nor did we observe differences in MOR binding between smokers and nonsmokers. Interestingly, we showed that in smokers MOR availability in bilateral superior temporal cortices during the placebo condition was negatively correlated with scores on the Fagerström Test for Nicotine Dependence (FTND). Also in smokers, smoking-induced decreases in [^11^C]carfentanil binding in frontal cortical regions were associated with self-reports of cigarette liking and wanting. Although we did not show differences between smokers and nonsmokers, the negative correlation with FTND corroborates the role of MORs in superior temporal cortices in nicotine addiction and provides preliminary evidence of a role of endogenous opioid signaling in frontal cortex in nicotine reward.

## Introduction

Tobacco use is the largest preventable cause of death and disease in the United States. In 2011, 19% of adults (43.8 million) were current smokers [10]. The reinforcing effects of nicotine are mediated, in part, via its effects on α4β2 nicotinic acetylcholine receptors, which result in activation of dopamine (DA) neurons and increased release of DA in the nucleus accumbens (NAc) [11]. The ability of most drugs of abuse to increase DA in the NAc, is believed to be a common mechanism through which drugs of abuse exert their reinforcing effects [41]. Specifically, acute nicotine has been shown to change met-enkephalin in striatum in ways that are interpreted to indicate that nicotine enhances the release and synthesis of met-enkephalin in striatum [20]. However, preclinical studies have also shown that nicotine increases release of endogenous opioids [15]. These effects are likely to contribute to nicotine's reinforcing effects because nicotine is not reinforcing in knockout mice that do not express mu opioid receptors (MORs) [6]. Adaptations in endogenous opioids secondary to chronic smoking are also likely to contribute to the addictiveness of nicotine. Indeed, repeated nicotine administration results in increased expression of MORs [46]. Moreover, naloxone, a MOR antagonist drug, can trigger withdrawal in animals exposed chronically to nicotine [35] and in daily smokers [28]. Further, polymorphic variants in the mu receptor (Asn40Asp variant) predict response to nicotine replacement therapy [31]. Thus, understanding the acute and long-term effects of nicotine on the opioid system in humans might provide better strategies for the development of treatment medications for nicotine dependence.

In this study, we tested the hypothesis that nicotine at doses delivered through a cigarette increases the release of endogenous opioids in the human brain and that chronic smokers exhibit neuroadaptations in mu receptors. We assessed the effects of smoking a cigarette on the binding of the mu-opioid agonist receptor radioligand [^11^C]carfentanil using positron emission tomography (PET) and compared the responses in nonsmokers to those in smokers. [^11^C]carfentanil binding in the brain is sensitive to competition with endogenous opioids [49], and thus we hypothesized that its binding would be decreased after smoking a cigarette. We also hypothesized that chronic smokers would show decreased endogenous opioid release when not under the effects of nicotine and thus would have increases in MOR when tested at baseline.

## Materials and Methods

### Ethics Statement

The studies were approved by the Johns Hopkins Medicine (JHM) Office of Human Subjects Research - Institutional Review Boards and the NIH Combined Neuroscience Institutional Review Board. After explaining the procedure, written informed consent was obtained from each subject.

### Subjects

Ten smokers and ten age-matched nonsmokers were recruited specifically for this study. The basic demographics and smoking-related measures are given in Table 1. Inclusion criteria for smokers were smoking 10–45 cigarettes per day for at least 2 years, urinary cotinine ≥200 ng/mL, and no desire to quit or reduce smoking. Inclusion criteria for nonsmokers were having smoked 1–20 cigarettes in their lifetime, no smoking in the past year, and urinary cotinine <30 ng/mL. Otherwise, inclusion criteria were the same for both groups: males and females 21–50 years old and estimated IQ ≥85. We followed the NIH policy for inclusion of men and women because we had no strong reasons to exclude by gender. Due to scan scheduling availability, female subjects were studied without controlling for menstrual cycle stage or use of birth control medication. Subjects were excluded if they had current or past psychiatric disorders (including drug abuse or dependence other than nicotine dependence), neurological diseases, significant medical illnesses, or those who were on psychoactive medications. Subjects were recruited using public advertisements, and were initially screened by phone, and subsequently evaluated for eligibility by a physician. As part of the screening procedure, subjects had a physical, psychiatric, and neurologic examination. They completed the Fagerström Test for Nicotine Dependence (FTND) [22], [3] and completed a neuropsychological battery to insure that they were not cognitively impaired. Routine laboratory tests were performed, including toxicology screening to rule out the use of common drugs of abuse. Subjects were instructed to abstain from alcohol and drugs (except caffeine, nicotine, and non-psychoactive prescription drugs) 24 hours before each session, and smokers abstained from smoking after 12:00 midnight the night before each session. Subjects were admitted overnight at the Johns Hopkins Hospital General Clinical Research Unit to insure that they did not smoke the night prior to the study. On the morning of the PET scans, subjects were provided with a low calorie breakfast and completed breathalyzer testing for alcohol and breath carbon monoxide (<10 parts per million) for smokers.

**Table 1:** Demographics of participants and radioligand information of placebo and active cigarette scans

Variables	Smokers	Nonsmokers
Demographics and smoking- and alcohol-related measures		
**Number of subjects (Sex)**	10 (8 M/2 F)	10 (6 M/4 F)
**Age (years)**	32.5±8.2 (range: 23–50)	34.3±10.7 (range: 22–50)
**Fagerström test for nicotine dependence**	6.8±1.8 (range: 5–10)	0
**Number of cigarettes per day**	19.5±12.7 (range: 10–45)	0
**Number of drinks per week**	1.2±1.7	1.3±1.6
**Drinking days per week**	0.7±1.0	0.8±1.1

Cigarette type for PET Sessions	Placebo	Active	Placebo	Active
Radioligand information				
**Injected radioactivity (MBq)**	666+18.5	654.9+48.1	662.3+25.9	680.8+25.9
**non-radioactive mass (µg)**	0.98±0.41	1.22±0.54	1.29±0.60	1.15±0.57
Specific activity (MBq per mole)	349.6+88.0	329.3+75.6	273.5+94.0	285.0+133.6

Values are mean ± standard deviation. In demographics, M stands for males, and F, for females.

### PET imaging

The subjects received two PET scans, each on a separate day with [^11^C]carfentanil. No statistical differences were noted in injected radioactivity, non-radioactive mass, and specific activity (Table 1) between placebo- and active-cigarette scans for smokers and non-smokers, or between smokers and non-smokers in the placebo- and active-cigarette scans. PET studies were performed on the High Resolution Research Tomograph (HRRT, CPS Innovations, Inc., Knoxville, TN). In preparation for the study, two intravenous catheters were placed, one for radiotracer injection and the other for blood withdrawal to measure nicotine concentrations in plasma (immediately after smoking, every 5 min for 15 min, then every 10 min until the PET scan was completed). Prior to each PET scan, subjects smoked either a placebo or a nicotine-containing cigarette (see below) and within 10 min after completion of smoking underwent dynamic PET scanning. Dynamic scans were obtained using three-dimensional list mode acquisition for 80 min following the intravenous bolus injection of [^11^C]carfentanil. A 6-min transmission scan was acquired prior to each dynamic scan using a rotating Cs-137 source for attenuation correction. A custom-made thermoplastic mask was employed to reduce head motion during the PET data acquisition times. [^11^C]carfentanil was synthesized via the reaction of [^11^C]methyliodide and a nor-methyl precursor as previously described [14] and was injected via a venous catheter.

### Cigarette smoking procedure

Subjects smoked either a Quest 1 cigarette (active, 0.6 mg nicotine) or a Quest 3 cigarette (placebo, <0.05 mg nicotine) before each PET session in a portable smoking booth attached to the ventilation system of a room adjacent to the scanner. Subjects took 8 puffs over a 10-min period using a CReSS smoking topography system (Plowshares, Inc., Baltimore, MD) to approximate equivalent smoking topography. The order of placebo and active cigarette conditions was counterbalanced across subjects.

### Self-report Measures

Subjects were administered the Minnesota Nicotine Withdrawal Scale (MNWS [24]) and the Tobacco Craving Questionnaire-Short Form (TCQ-SF) [23] before and after each PET scan session. The following Visual Analog Scale (VAS) items assessed the effects of the cigarette “right now”: feel the effect, good effect, bad effect, like the effect, and want a cigarette. Subjects verbally rated each item on a scale from 0 to 10. VAS items were completed at baseline and at 5, 10, 15, 20, 25, 35, 40, 50, 60, 70, 80, and 90 min post-smoking.

### Measurement of Nicotine Concentrations in Plasma

Blood specimens (5 mL) were collected in 7-mL green-topped Vacutainer tubes containing lithium oxalate, and immediately placed on ice. Specimens were centrifuged within 1 h and 1.0 mL aliquots of plasma stored in cryotubes at −80°C until analysis. Nicotine, cotinine, trans-3′-hydroxycotinine (OH-cotinine) and norcotinine were measured concurrently in 0.5 mL plasma specimens by a previously validated liquid chromatography tandem mass spectrometry (LCMSMS) method [21]. Briefly, 2 mL 0.1% formic acid were added to plasma specimens and centrifuged at 4,000×g for 5 min at 4°C. Supernatants were submitted to solid phase extraction with Strata-XC cartridges (Phenomenex, San Jose, CA), with final elution in 3% NH_4_OH in methanol. LCMSMS analysis was performed with a Shimadzu liquid chromatography system (Shimadzu Corporation, Columbia, MD, USA), a Synergi Polar-RP 100A interfaced to a 3200 QTrap (AB Sciex, Foster City, CA, USA) with a Turbo V ESI source. Standard mobile phases were used with gradient elution and a total run time of 12 min. Mass spectrometric data were acquired in positive electrospray ionization mode and multiple reaction monitoring mode (MRM). The following transitions were monitored (quantification transition in bold): 163.2>132.2 and 163.2>84.2 for nicotine; 177.2>80.1 and 177.2>98.1 for cotinine; 193.2>80.2 and 193.2>134 for OH-Cotinine; 163.2>80.2 and 163.2>118.2 for norcotinine; 167.2>136.1 and 167.2>121 for Nicotine-d_4_; 180.2>80.2 and 180.2>101.2 for Cotinine-d_3_; 196.2>79.9 and 196.2>134.1 for OH-Cotinine-d_3_; and 167.2>84.2 and 167.2>139.2 for norcotinine-d_4_. Linearity ranges with 1/x weighting for nicotine and 1/x^2^ for metabolites were 1 to 500 ng/mL for cotinine, OH-cotinine and norcotinine, and from 2.5 to 500 ng/mL for nicotine. Assay accuracy at low, medium and high QCs was 90.1–103.5% (n = 20) and imprecision was 4–13.8% (n = 20).

### *Reconstruction of PET data*

Emission PET scans were reconstructed using the iterative ordered-subset expectation-maximization algorithm correcting for attenuation, scatter, and dead-time [42]. The radioactivity was corrected for physical decay to the injection time and re-binned to 30 dynamic PET frames of 256 (left-to-right) by 256 (nasion-to-inion) by 207 (neck-to-cranium) voxels. The frame schedules were six 30 s, seven 60 s, five 120 s, and twelve 300 s frames. The final spatial resolution is expected to be less than 2.5 mm full-width at half-maximum in three directions [45].

### MRI acquisition

On a separate occasion, a spoiled gradient (SPGR) sequence 1.5 or 3 T MRI was obtained on each subject for anatomical identification of the structures of interest using the following parameters: Repetition time, 35 ms; echo time, 6 ms; flip angle, 458; slice thickness, 1.5 mm with no gap; field of view, 24×18 cm^2^; image acquisition matrix, 256×192, reformatted to 256×256 for the 1.5 T. Repetition time, 2110 ms; echo time, 2.73 ms; flip angle, 8; slice thickness, 0.8 mm with no gap; field of view, 24×18 cm^2^; image acquisition matrix, 320×288, reformatted to 256×256 for the 3 T.

### PET data analysis

#### Volumes of interest (VOIs)

VOIs were manually defined for putamen (Pu), caudate nucleus (CN), hippocampus (HP), and cerebellum (Cb) using locally developed VOI tool (VOILand). Striatal VOIs were divided into ventral striatum (vS) and anterior or posterior dorsal subdivisions by the anterior-commissure plane, as previously described [5], [37], [39]. Other subcortical VOIs, including globus pallidus (GP), thalamus (Th), and amygdala (Am) were defined with FIRST software [40] and manually adjusted on individual MRIs. Cortical VOIs were automatically defined using Freesurfer [19] software including subdivisions of frontal (Fr), temporal (Tp), parietal (Pa), and occipital (Oc) cortices, fusiform gyrus (Fs), cingulate (Cg), and insula (In). VOIs for In and Oc were manually adjusted on individual MRIs. The Oc VOI included cuneus, lingual, lateraloccipital, and pericalcarine lobules given by Freesurfer, and served as reference region, after confirming that these regions showed similarly low radioactivity. A total of 78 VOIs were transferred from MRI to PET spaces using MRI-to-PET coregistration parameters given by the SPM5 coregistration module [2], [33] to obtain time-activity curves (TACs) of regions.

#### Derivation of PET outcome variables

A set of reference tissue methods were employed to obtain the binding potentials (BP_ND_) [27] of regions (reference region  =  Oc), including the reference region graphical analysis (RTGA) [32] with k_2_^R^ (the brain-to-blood efflux rate constant of Oc) set at 0.104 min^−1^ [17], multilinear reference region method with 2 parameters (MRTM2) [26], and the bolus-plus-infusion transformation of bolus-only scans [29]. Images of BP_ND_ were generated by the three methods and transferred to the Montreal Neurological Institute (MNI) standard space applying parameters of PET-to-MRI coregistration (See above) and spatial normalization given by SPM unified segmentation method [1] in one step, and smoothed by a Gaussian kernel (8 mm Full with at half maximum) to submit to SPM5 statistical methods.

#### Voxel-wise statistical analysis

SPM5 was used to examine group differences and correlations of [^11^C]carfentanil BP_ND_ to smoking status measures, and visual analogue scales on smoking, as described in the results section. A significance level of p<0.001, uncorrected was employed for SPM analyses with the cluster volume threshold set at 0.4 mL (k>50). Locally prepared gray matter probability, tissue classification, and Brodmann area maps were used for anatomical identification and visualization of SPM clusters. Briefly, Freesurfer-derived brain region VOIs of 399 subjects (Age range: 18–40 years) were transferred to the SPM standard space using the SPM unified segmentation method [1] to generate probability maps of individual structures. The gray matter probability map was generated by summing individual gray matter probability maps to visually confirm whether clusters fell within gray matter areas. The tissue classification map was generated by assigning voxels to brain structures, starting from the largest (white matter) to the smallest (the ventral striatum) structures with a probability threshold of 0.2 to identify anatomical locations of peaks, and calculate structure compositions of clusters. The Brodmann area map was prepared by spatially aligning a published atlas [34] to the local standard brain to report Brodmann areas that were closest to the peaks and within the clusters, if any.

## Results

**Figure 1:**
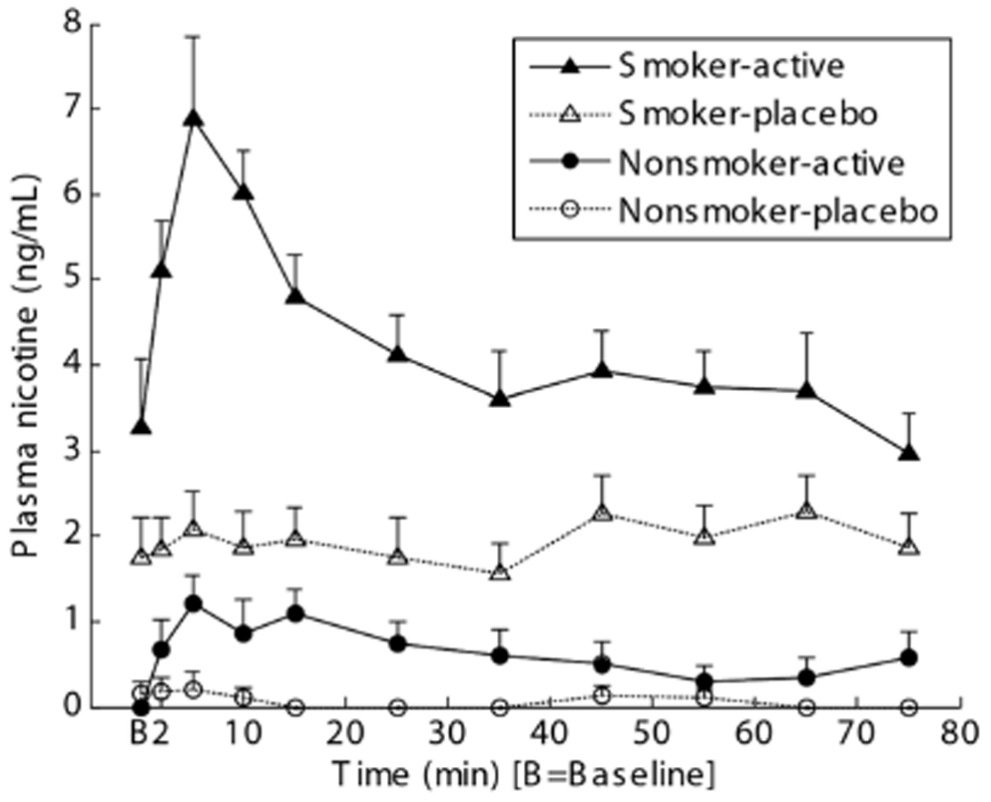
Line plots of mean concentrations across subjects of nicotine in plasma versus time of active and placebo cigarette scans. PET data acquisition began between 5 and 10 min on the time axis in individual subjects.

None of participants fulfilled criteria of alcohol abuse or dependence, and no statistical differences were noted regarding alcohol-related metrics between smokers and nonsmokers (Table 1). For smokers, plots of mean plasma nicotine concentration peaked at 5 min and reached a plateau around 30 min in the active cigarette scan, but remained around the baseline level throughout the placebo cigarette scan (Fig. 1). The active cigarette scan condition showed higher plasma nicotine levels than the placebo cigarette scan condition (Table 2), while no statistical differences were observed between the two conditions for its metabolites. For nonsmokers, concentrations increased initially and declined slowly thereafter (Fig. 1). Plasma nicotine concentrations were substantially greater for smokers than nonsmokers for both cigarette conditions (Table 2).

**Table 2:** Concentrations of nicotine and metabolites in plasma during active and placebo cigarette scans.

Variables	Smokers		Nonsmokers	
Cigarette type for PET Sessions	Placebo	Active	Placebo	Active
Nicotine (ng/mL)	1.9±0.9#	4.4±1.4* #	0.1±0.1	0.7±0.7
Nicotine (2–10 min; ng/mL)	1.9±1.2#	5.8±1.9* #	0.2±0.4	0.9±0.9
Cotinine (ng/mL)	217±106	230±97	<1	<1
Trans-3-hydroxy-cotinine (ng/mL)	70.5±38.7	63.6±38.6	0	0
Norcotinine (ng/mL)	2.6±2.3	3.2±1.9	0	0

Mean ± standard deviation (ng/mL) of individual subjects' means across 2–75 min, except for the second nicotine row representing nicotine concentrations only from 2–10 min.

Limits of quantification were 1 ng/mL for cotinine, OH-cotinine and norcotinine, and 2.5 ng/mL for nicotine at individual time point.

* Active cigarette scan values > placebo cigarette scan value at p<0.01; paired t-test.

# Smoker > nonsmoker at p<0.00001; t-test.

PET data were examined first for pharmacological effects (i.e., not including behavioral effects) of smoking active cigarettes on [^11^C]carfentanil binding. Of 78 VOIs, the right parahippocampus alone showed a significant decrease of [^11^C]carfentanil BP_ND_ in the active-cigarette scan (t = -5.96; p = 0.0002; df = 9; paired t-test) in smokers. However, this region showed relatively low [^11^C]carfentanil BP_ND_ (0.21±0.06 vs. 0.25±0.06 (mean ± SD; unitless) for placebo and active scans, respectively). Other regions did not show changes in [^11^C]carfentanil BP_ND_ between the two conditions separately for smokers and nonsmokers. The voxel-wise tests (SPM) showed no changes of [^11^C]carfentanil BP_ND_ between two conditions for smokers as well as for nonsmokers despite significant differences in plasma nicotine concentrations. Magnitudes of changes of [^11^C]carfentanil BP_ND_ between the two conditions remained undistinguishable between the two groups. No correlations of [^11^C]carfentanil BP_ND_ to plasma nicotine concentrations were observed including all plasma data points (2–75 min) or plasma samples around the peak (i.e., using 2, 5, and 10 min data) alone.

**Figure 2:**
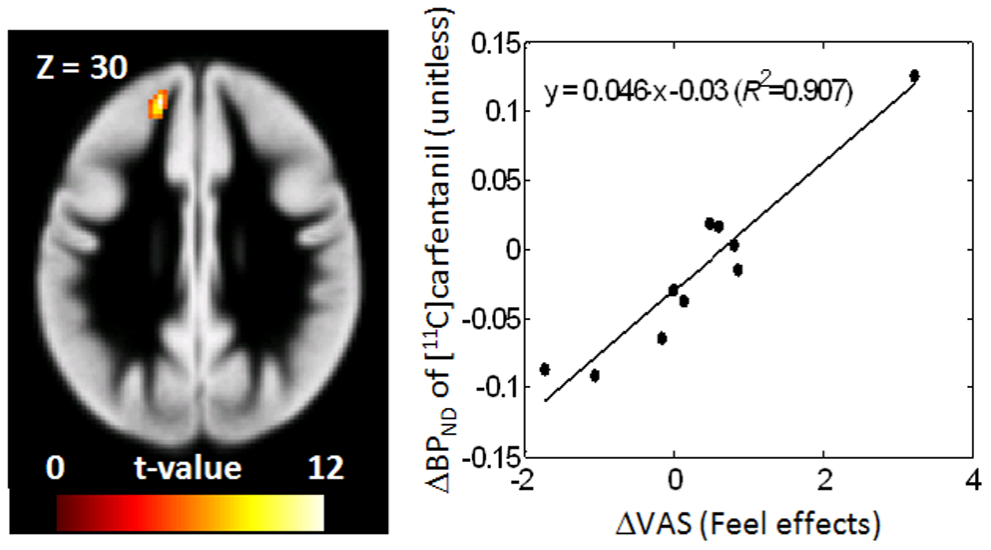
Positive correlation clusters of Δ[^11^C]carfentanil binding potential (BP_ND_) (placebo - active) versus ΔVAS of feel the effect category in smokers, displayed on trans-axial images of a gray-matter probability maps. Scatter plots of cluster Δ[^11^C]carfentanil BP_ND_ values to ΔVAS are shown together with regression lines. VAS stands for the visual analog scale of smoking effects, and *R^2^* stands for the coefficient of determination of linear regression.

Correlations of changes of [^11^C]carfentanil BP_ND_ (Δ[^11^C]carfentanil BP_ND_, defined as placebo-cigarette scan values minus active-cigarette scan values) with changes of self-reported VAS items (ΔVAS, defined as active minus placebo using means of data recorded between 20 and 80 min after the tracer injection) were examined. Smokers had single positive correlation clusters in left rostral frontal lobe for VAS items feel and like the effect, and good effect (Table 3 and Fig. 2). The three clusters spatially overlapped each other substantially. These correlations remained statistically significant (the coefficient of determination, *R^2^*>0.704) after removing one subject who showed larger ΔVAS values than other smokers in all three scores. The three clusters should be considered to represent one cluster of indistinguishable contributions from these three ΔVAS categories because the three ΔVAS categories were mutually correlated (*R^2^*>0.77; p<0.0001). Smokers also showed one cluster in right rostral frontal cortex for the VAS want a cigarette, although the cluster volume (0.24 mL) did not reach the set criterion (0.4 mL). SPM correlation analyses were not performed on nonsmokers because ΔVAS values were skewed heavily around 0 for this group. VOI-based analysis did not identify Δ[^11^C]carfentanil BP_ND_, - ΔVAS correlations in any regions in smokers. No correlations were noted for nonsmokers in SPM and VOI-based analyses.

**Table 3:** Clusters of Δ[^11^C]carfentanil BP_ND_ (placebo - active) to ΔVAS (active - placebo) correlation.

VAS	Peak coordinates	Peak t-values	Cluster volumes	Anatomical descriptions
Positive correlations				
Feel effect	−16 48 30	13.07	0.83 mL	Lt. rostral frontal lobe (53.9%) White matter (38.5%)
Good effect	−16 48 30	10.86	0.57 mL	Lt. rostral frontal lobe (43.7%) White matter (45.1%)
Like effect	−18 48 26	10.05	1.14 mL	Lt. rostral frontal lobe (71.8%) White matter (22.5%)

Significance criteria: p<0.001, uncorrected and volume >0.4 mL.

Percentages in the last column indicate anatomical constituents of clusters.

We also compared [^11^C]carfentanil BP_ND_ for the placebo and for the differences between placebo and active cigarette scans between smokers and nonsmokers. No regions showed differences in VOI-based analyses, and no differences in clusters were identified in voxel-wise tests separately for active- and placebo-cigarette scans.

**Figure 3:**
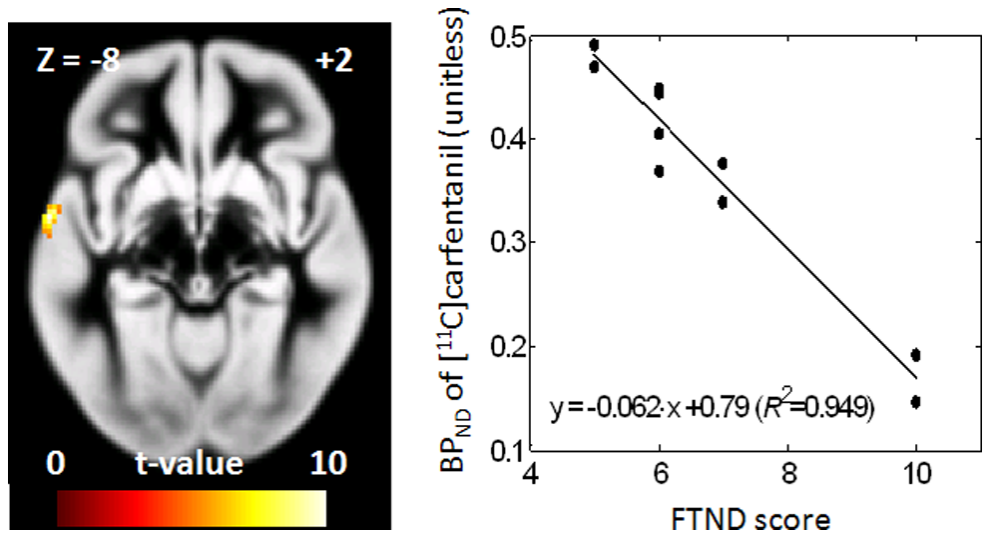
Correlation clusters of [^11^C]carfentanil binding potential (BP_ND_) of placebo-cigarette scans versus the Fagerström Test for Nicotine Dependence (FTND) in smokers, displayed on trans-axial images of a gray-matter probability maps. Right panels show scatter plots using cluster [^11^C]carfentanil BP_ND_, together with regression line. In regression equations, *R^2^* stands for the coefficient of determination.

Finally, the following exploratory tests were performed on smokers alone. Correlation analysis between [^11^C]carfentanil BP_ND_ and FTND showed symmetrical negative correlation in superior temporal lobes (Table 4 and Fig. 3). A left-side negative correlation cluster also was observed for [^11^C]carfentanil BP_ND_ to current smoking status (cigarettes per day, CPD). The right side cluster did not reach the set significance criteria for this correlation. Spatial agreement of correlation clusters between the two smoking measures may be explained by the observation that these measures were highly correlated (CPD  = 6.7•FTND - 26.2; R^2^ = 0.909). A positive correlation of lesser significance and volume was observed in left precentral gyrus with CPD.

**Table 4:** Clusters of placebo-cigarette [^11^C]carfentanil BP_ND_ to current nicotine-dependence and smoking statuses correlations in smokers.

N/P	Peak coordinates	Peak t-values	Cluster volumes	Anatomical descriptions
Placebo-cigarette scan [^11^C]carfentanil BP_ND_ vs. FTDN				
N	−62 −2 −8	10.31	1.01 mL	Lt. superior temporal lobe (73.5%) Lt. precentral gyrus (22.1%)
N	62 4 2	7.47	0.54 mL	Rt. superior temporal lobe (62.5%) Rt. precentral gyrus (29.2%)

Placebo-cigarette scan [^11^C]carfentanil BP_ND_ vs. cigarette per day				
N	−64 −2 −4	14.25	0.97 mL	Lt. superior temporal lobe (68.3%) Lt. precentral gyrus (31.7%)
P	−46 −14 42	6.81	0.48 mL	Lt. precentral gyrus (76.9%) Lt. postcentral gyrus (19.2%)

Significance criteria: p<0.001, uncorrected and volume>0.4 mL

P and N in the first column stand for clusters of positive and negative correlations, respectively.

Percentages in the last column indicate anatomical constituents of clusters.

## Discussion

Active cigarettes in this study were expected to cause nearly 50% occupancy of nicotinic acetylcholine receptors (nAChRs) across brain regions (e.g., [18]), whereas Brody et al. [7] reported 26% and 79% occupancy of nACHRs by Quest 1 and 3 cigarettes, respectively. Although similar levels of occupancies of nAChRs were expected, no regions showed changes of [^11^C]carfentanil BP_ND_ in this study. This finding conflicts with results of a similar study [44] that reported clusters of decreased [^11^C]carfentanil BP_ND_ in the anterior cingulate and three increased clusters in the left amygdala, left ventral striatum, and right thalamus in the active-cigarette (1.01 mg nicotine/cigarette) condition compared to the placebo cigarette (0.08 mg nicotine/cigarette) condition. This discrepancy is likely to reflect the much lower nicotine concentrations achieved in our study (three fold lower plasma nicotine concentrations) and could be interpreted to suggest that higher nicotine doses than the ones achieved in our study (peak plasma level of 7 ng/mL versus average levels of 18 ng/mL in Scott et al. [44] might be necessary for endogenous opioid release. Differences in peak plasma nicotine concentration between smokers and nonsmokers were likely due to minimal inhalation on the part of nonsmokers. However, the differences in PET methodology (two 90-min scans on separate days in this study versus one 90-min scan for two cigarette sessions in one day) might also contribute to the differences in findings. Using the same Quest cigarettes as this study, Ray et al. [43] reported no changes in [^11^C]carfentanil BP_ND_ between the two cigarette conditions (see below), although they analyzed smokers of A/A genotype carriers of OPRM1 A118G genotype and G allele carriers separately. Thus, further studies are needed to investigate the pharmacological effects of nicotine in endogenous opioid release, including its potential modulation by OPRM1 A118G genotypes.

This and aforementioned studies [43], [44] identified different clusters of (Δ[^11^C]carfentanil BP_ND_ to ΔVAS correlations). Scott et al. [44] identified one cluster in the thalamus for the 'alert' score alone (i.e., the study did not identify correlations in VAS categories used in our study). Ray et al. [43] reported positive association of [^11^C]carfentanil BP_ND_ with changes of self-reported nicotine reward in right amygdala, caudate nucleus, anterior cingulate cortex, and thalamus using VOI-based analysis. Interestingly, the correlation was found in G allele carrier smokers (OPRM1 A118G genotype), but not in A/A genotype carriers. The closest equivalent to the reward measures in Ray et al. [43] would be the VAS items like the effect and good effect in our study for which the correlation was found in the left rostral frontal lobe (corresponding to Brodmann area 10), in the mixed genotype population. Moreover, positive correlations of cerebral blood flow changes to the amount of monetary rewards (suggestive of positive effects) were reported in a vicinity of current Δ[^11^C]carfentanil BP_ND_ to ΔVAS correlations ([x, y, z]  = −20 12 42 [36]) in smokers, but not in nonsmokers. It is intriguing to speculate that the left rostral frontal lobe cluster (Fig. 2) might be indicative of the dopamine-opioid interaction because the hedonic effects of cigarettes, such as euphoria and craving, are associated with dopamine discharge in the striatum (e.g., [4]). Regarding the dopamine-opioid interaction, Colasanti et al. [12] demonstrated decreases of [^11^C]carfentanil BP_ND_ in multiple brain regions in the high dose scan (0.5 mg/kg) compared to a low dose (0.017 mg/kg) of oral amphetamine in healthy male nonsmokers. Because of no known direct actions of amphetamine on opioid neurotransmission and established amphetamine-induced dopamine release, the study suggested that dopamine-opioid interaction underpin the change of [^11^C]carfentanil BP_ND_. Visual inspection (Fig. 3) of SPM clusters and tabulated results (Table S3) of Colasanti et al. [12] suggested the left middle frontal gyrus cluster (volume: 816 voxels or 65.3 mL) span the positive correlation cluster in the left rostral frontal lobe we observed for the feel/like/good effect ΔVAS categories. Therefore, this cluster might be related to the potential dopamine-opioid interaction. Interestingly, the location in the rostral frontal region corresponds to a region where functional connectivity was positively correlated with decreases in withdrawal symptoms with nicotine replacement therapy in abstinent smokers [13]. Since dysphoria is a central symptom in nicotine withdrawal [25], this also implicates endogenous opioid signaling in medial prefrontal regions in the reversal of negative symptoms by nicotine replacement.

Smoker versus nonsmoker differences in [^11^C]carfentanil BP_ND_ should be discussed within the limitation that subjects received either active or placebo cigarettes in each scan. Although the current study identified no differences in VOI-based (except in parahippocampal gyrus but not corroborated by SPM) and in voxel-wise statistical tests, Scott et al. [44] demonstrated robust (>13%) differences (smokers < nonsmokers) in the rostral anterior cingulate, thalamus, nucleus accumbens, and amygdala. Ray et al. [43] did not examine smoker versus nonsmoker differences. Correlation analysis of [^11^C]carfentanil BP_ND_ with nicotine dependence and current smoking status measures in our study should also be interpreted with caution because those data were obtained under the placebo cigarette condition. Numerous studies have documented the expectancy and/or sensorimotor effects of denicotinized cigarettes in reducing tobacco deprivation-induced withdrawal symptoms [8], [9], [16]. Thus, expectancy effects might have contributed to the release of endogenous opioids in smokers and nonsmokers.

Here, we report correlations of [^11^C]carfentanil BP_ND_ with smoking two smoking metrics that though related represent different smoking-related properties. A recent paper [47] reported negative correlations of baseline (i.e., no-specific tasks) [^11^C]carfentanil BP_ND_ to FTND scores in the cingulate cortex, thalamus, amygdala, and insula cortex, in alcohol-dependent subjects (n = 21) after partial correlations accounting for gender and recent drinking status. Although these correlations were observed in alcohol-dependent subjects [48], our study and the Weerts et al. [48] study indicated negative associations of [^11^C]carfentanil BP_ND_ to nicotine dependence/smoking status in selected brain regions, including superior temporal lobes. Involvement of these lobes in nicotine dependence was implicated in a number of studies. Interestingly, cue-induced changes in fMRI BOLD signal negatively correlated to FTND in the right superior temporal gyrus in a study involving 30 smokers with similar FTND scores to our study [38], suggesting that a decrease in MOR might underpin decreased BOLD response at least in this region. In a separate study, the left superior temporal gyrus alone showed cue-induced changes of BOLD signals [30] in abstaining smokers (7 hours), although correlations to FTND or CPD were not examined.

These findings need to be confirmed with a larger sample size. One limitation of our study was the possible psychological cues provided by smoking the placebo cigarette. The behaviors, the paraphernalia, and the environment associated with cigarette smoking, even in the absence of nicotine, likely contribute to the craving and reward of cigarette smoking in some smokers. Future studies without the cues of the smoking environment could control for this confounding influence.

In summary, smokers demonstrated correlations in [^11^C]carfentanil BP_ND_ with nicotine dependence and smoking status. This suggests the need for further investigation of the role of MOR in nicotine dependence.
